# The mediating roles of screen time and sleep duration in the association between extracurricular physical activity and visual acuity in primary school students

**DOI:** 10.3389/fpubh.2026.1883751

**Published:** 2026-09-03

**Authors:** Jilin Li, Yang Wang, Xuehui Zhang, Ye Chen, Li Xi

**Affiliations:** 1 Sports Institute of Hefei Normal University, Hefei, Anhui, China; 2 Department of General Education, Anhui Xinhua University, Hefei, Anhui, China

**Keywords:** extracurricular physical activity, primary school students, screen time, sleep duration, visual acuity

## Abstract

**Objective:**

This study aimed to examine the association between extracurricular physical activity (EPA) and visual acuity (VA) among primary school students in Macau and investigate the mediating roles of screen time (ST) and sleep duration (SD).

**Methods:**

A stratified random cluster sampling method was used to recruit primary school students in Macau (*n* = 2,098). Data were collected using questionnaire surveys. Pearson correlation analysis and structural equation modeling were conducted.

**Results:**

EPA, ST, SD, and VA were significantly correlated (*p* < 0.01). Mediation analysis identified a direct association between EPA and VA and indirect associations through ST and SD. The estimated total association was 0.129 (95% CI [0.099, 0.162]), and the total indirect association through ST and SD was 0.058 (95% CI [0.039, 0.082]), accounting for 44.96% of the estimated total association. The specific indirect associations through SD and ST were 0.033 (95% CI [0.020, 0.052]) and 0.022 (95% CI [0.010, 0.037]), accounting for 25.58 and 17.05% of the estimated total association, respectively. The sequential indirect association through ST and SD was statistically significant but small (0.003, 95% CI [0.001, 0.006]), accounting for 2.33% of the estimated total association.

**Conclusion:**

EPA was positively correlated with VA among primary school students, and statistically detectable indirect associations were observed through ST, SD, and the sequential ST–SD pathway.

## 1 Introduction

Reduced visual acuity (VA) among children, particularly those of school age, represents an increasingly important public health concern. The global prevalence of myopia among children and adolescents increased from 24.32% in 1990–2000 to 35.81% in 2020–2023 and is projected to reach 39.80%, affecting more than 740 million children and adolescents by 2050 (1). Although myopia is not synonymous with reduced VA, uncorrected myopia is an important contributor to reduced distance VA in school-aged populations. Reduced VA may adversely affect classroom participation, academic performance, daily functioning, psychosocial development, and quality of life, particularly when the underlying condition remains undetected or inadequately corrected (2, 3). These potential educational, functional, and long-term ocular consequences highlight the importance of identifying modifiable behavioral factors associated with VA during childhood.

Reduced VA is a functional finding rather than a specific ophthalmologic diagnosis. In the general pediatric population, common causes include uncorrected refractive errors, such as myopia, hyperopia, and astigmatism, as well as amblyopia and strabismus. Less common causes include corneal, lenticular, retinal, optic nerve, and other organic ocular disorders (4). Among school-aged children in East Asia, myopia is particularly common and represents an epidemiologically important potential cause of reduced distance VA (5). Primary school students in Macao are situated within this regional context; however, reduced VA in this population should not be assumed to represent myopia in every case. The present study assessed VA rather than refractive status and did not use cycloplegic refraction, spherical equivalent refraction, or axial length to diagnose myopia. Therefore, myopia is discussed as one plausible contributor to reduced VA rather than as the directly measured outcome.

Several potentially modifiable lifestyle behaviors have been associated with VA or refractive outcomes in children, including physical activity, outdoor exposure, screen time, and sleep duration (6). Among these factors, extracurricular physical activity (EPA) may represent a potentially protective behavior (7). Each additional hour spent outdoors per week has been associated with an approximately 2% reduction in the odds of myopia among children and adolescents (8). Nevertheless, physical activity and outdoor exposure are not interchangeable. The association between physical activity and VA may partly reflect increased exposure to outdoor light and reduced time spent on sustained near-work activities rather than the intensity of physical activity alone (9). By contrast, prolonged screen time (ST) may increase exposure to short viewing distances and sustained near-work and may simultaneously displace outdoor and physically active behaviors (10). Each additional hour of daily digital screen use was associated with a 21% increase in the odds of myopia, although substantial heterogeneity and the predominantly observational evidence warrant cautious interpretation (11). Higher physical activity has also been associated with lower ST (12), suggesting that the allocation of time to screen-based behavior may be statistically involved in the association between EPA and VA. However, educational, recreational, and passive screen exposure may differ in viewing distance, duration, continuity, and visual demand. Because the present study assessed total ST, it was not possible to distinguish these screen-use categories.

Sleep duration (SD) has also been investigated as a potential correlate of children’s ocular and visual functioning (13). Screen exposure, particularly when occurring near bedtime, may delay sleep onset or displace time otherwise available for sleep, indicating an interrelationship between ST and SD (14). However, evidence regarding the relationship between sleep duration and myopia or VA remains inconsistent. No statistically significant pooled association has been observed between sleep duration and myopia prevalence, suggesting that sleep dimensions other than duration warrant further investigation (15). Consequently, SD should be considered a potentially relevant behavioral correlate rather than an established independent determinant of VA.

Several biological and behavioral pathways may contribute to the observed relationships among EPA, ST, SD, and VA. First, bright outdoor light may stimulate retinal dopamine signaling, which has been proposed to regulate ocular growth and inhibit excessive axial elongation associated with the development of myopia (16). Second, prolonged screen-based near-work may involve sustained accommodation, short viewing distances, reduced blink frequency, and increased visual fatigue, while simultaneously reducing opportunities for outdoor exposure. Third, screen use may interfere with sleep through time displacement, psychological stimulation, delayed bedtimes, and disruption of regular circadian routines. In contrast, regular physical activity may support sleep regulation through energy expenditure and circadian entrainment (17, 18). A further proposed pathway involves metabolic regulation. Sedentary behavior and insufficient physical activity may be associated with poorer insulin sensitivity, while hyperinsulinemia and insulin resistance have been hypothesized to affect ocular growth through insulin- and insulin-like growth factor-related signaling pathways (19). Nevertheless, the proposed relationship between insulin resistance and myopia remains hypothetical, and insulin resistance was not measured in the present study. It was therefore not included as a variable in the hypothesized mediation model.

The 24-h movement behavior framework conceptualizes physical activity, sedentary behavior, and sleep as interdependent components of a finite daily period rather than as isolated exposures (20). Time allocated to one behavior necessarily affects the time available for other behaviors, and these behavioral patterns may vary according to individual, family, school, and broader environmental contexts (21). From this perspective, greater participation in EPA may be associated with lower ST and more favorable sleep patterns, which may, in turn, be associated with VA. Nevertheless, most previous studies have examined these behaviors separately, and limited evidence is available regarding the individual and sequential mediating roles of ST and SD in the association between EPA and VA, particularly among primary school students in Macao. Therefore, the present study aimed to examine the association between EPA and VA and to investigate the individual and sequential mediating roles of ST and SD in this association. In the present study, mediation refers to statistically estimated indirect effects within the hypothesized model. Given the cross-sectional design, these effects should not be interpreted as evidence of temporal ordering or causality.

## 2 Literature review and research hypothesis

Previous studies have reported associations between EPA and children’s visual outcomes. Higher levels of physical activity have been associated with a lower probability of visual impairment or reduced VA (22), particularly when activities occur outdoors and provide greater exposure to natural light (23). Physically active children may also spend less time engaging in sustained near-work activities, such as prolonged reading and screen use, thereby potentially reducing visual strain (9). However, the findings have not been entirely consistent. In one recent study, meeting the PA guideline alone was not significantly associated with myopia (aOR = 0.87, 95% CI 0.74–1.01), whereas meeting both the PA and ST guidelines was associated with lower odds of myopia (aOR = 0.73, 95% CI 0.56–0.97) (24, 25). These inconsistencies may reflect differences in activity type, intensity, duration, outdoor setting, measurement method, age group, educational demands, and neighborhood or school environment. In particular, participation in indoor physical activity may not provide the same light exposure as outdoor activity. Therefore, an observed association between EPA and VA may reflect a combination of physical activity, outdoor exposure, and displacement of near-work rather than an independent effect of exercise intensity. The relationship should consequently be interpreted within the broader pattern of children’s daily behavior rather than as a simple direct protective effect.

EPA, ST, and VA are behaviorally interrelated (26). ST frequently involves sedentary behavior and near-work exposure, although the visual demands of educational computer use, recreational gaming, video viewing, and passive screen exposure may differ. Participation in EPA occupies time that might otherwise be allocated to sedentary or screen-based activities (27). This time-use substitution pattern is consistent with evidence that participation in extracurricular activities is associated with lower recreational ST (12). Within the 24-h movement behavior framework, EPA and ST should therefore be regarded as interdependent components of daily time use rather than as completely independent exposures (20). Within the 24-h movement behavior framework, EPA, ST, and SD represent interdependent components of children’s daily time use. Greater participation in EPA may reduce the time available for screen-based sedentary activities, while lower ST may be associated with longer or more regular sleep. These behavioral associations provide a theoretical basis for examining both the individual mediating roles of ST and SD and their sequential mediating role in the association between EPA and VA. However, because these variables were measured concurrently, the proposed sequence represents a theoretically specified mediation model rather than a confirmed temporal process.

Sleep is another component of the 24-h movement behavior system and may be related to visual functioning through physiological recovery, circadian regulation, and the organization of children’s daily activities (28). Sleep may contribute to recovery from sustained visual demands, whereas insufficient or irregular sleep may coexist with visual fatigue and less favorable ocular functioning (29, 30). At the behavioral level, physical activity has been associated with more favorable sleep outcomes (31). At the same time, greater ST has been associated with shorter sleep duration; for example, video viewing was inversely associated with sleep duration (*b* = −0.07, 95% CI − 0.101 to −0.044) among children aged 9–10 years (14).

SD represents only one dimension of sleep. Sleep quality, sleep fragmentation, bedtime, chronotype, and regularity may provide additional information that cannot be captured by duration alone. Moreover, current evidence on the association between SD and myopia remains heterogeneous and inconclusive, with a pooled estimate showing no statistically significant association between sleep duration and myopia prevalence (OR = 0.905, 95% CI 0.782–1.047) (15). From the perspectives of time use and behavioral organization, EPA, ST, and SD occur within limited temporal resources and may jointly correspond to variations in recovery, development, cognitive functioning, and VA (32). Therefore, the proposed sequence should be considered a theoretically informed statistical pathway rather than a confirmed biological or temporal process.

This study aimed to examine the association between EPA and VA among primary school students in Macao and to investigate the individual and sequential mediating roles of ST and SD in this association. Based on the 24-h movement behavior framework, time-use substitution theory, and the available empirical evidence, the following hypotheses were proposed:

*H1*: EPA significantly and positively predicts VA.

*H2*: ST mediates the relationship between EPA and VA.

*H3*: SD mediates the relationship between EPA and VA.

*H4*: ST and SD jointly play a chain mediating role in the relationship between EPA and VA.

**Figure 1:**
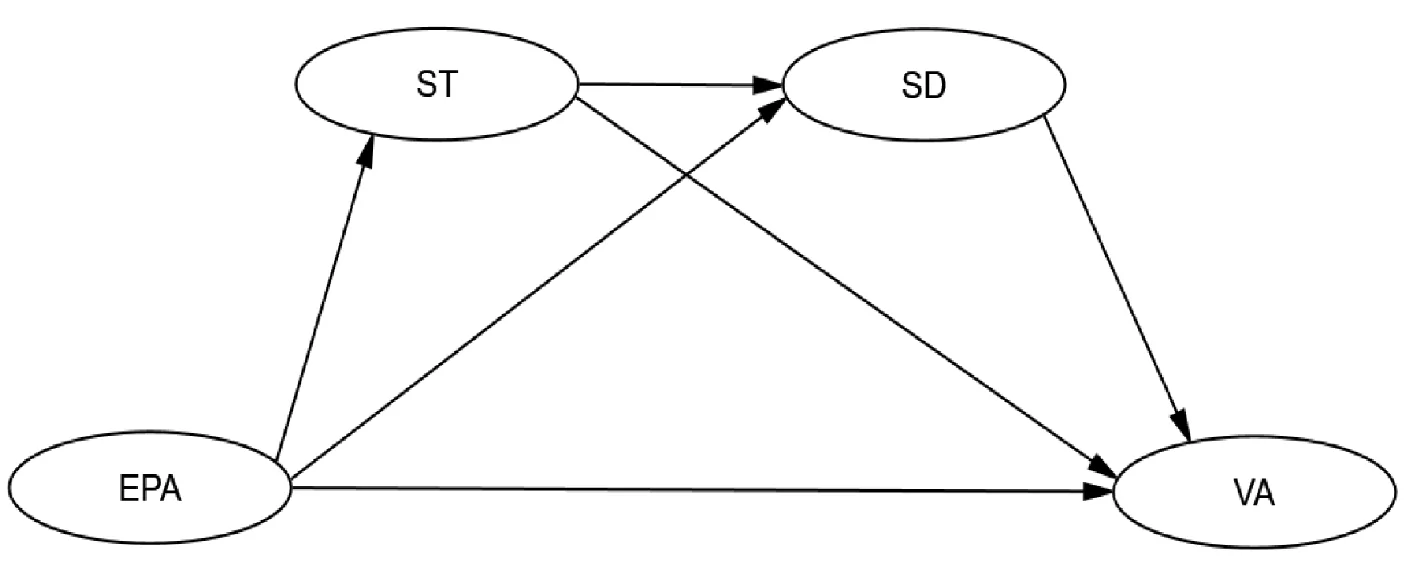
Hypothesized directed acyclic graph (DAG) among EPA, ST, SD, and VA. EPA, extracurricular physical activity; ST, screen time; SD, sleep duration; VA, visual acuity.

Based on these hypotheses, a hypothesized directed acyclic graph (DAG) was constructed to represent the proposed relationships among EPA, ST, SD, and VA, as shown in Figure 1.

## 3 Materials and methods

### 3.1 Study design and setting

This cross-sectional study was based on data from the 4th Macao Citizen Physical Fitness Surveillance, conducted between July and November 2020 by the Sports Bureau of the Macao Special Administrative Region in collaboration with the China Institute of Sport Science. Macao is a highly urbanized Special Administrative Region of China. In 2020, Macao had a resident population of approximately 6,83,100 (33). Approximately 36,220 students were enrolled in primary education during the corresponding academic year (34). These demographic and educational characteristics provide contextual information on the underlying population from which the surveillance sample was drawn.

The original surveillance program was approved by the Ethics Committee of the China Institute of Sport Science on June 7, 2019 (approval no. CISSLA-20190607), and written informed consent was obtained from the participants’ parents or legal guardians. The present secondary analysis was subsequently approved by the Research Ethics Committee of Hefei Normal University (approval no. 2024-LLSC-030).

The target population comprised students enrolled in Grades 1–6 at primary schools in Macao. The source population for the present analysis comprised primary-school participants in the 4th Macao Citizen Physical Fitness Surveillance. A stratified multistage cluster sampling procedure was used. Schools were first stratified according to geographical location, after which schools were randomly selected within each stratum. Classes within the selected schools were treated as clusters, and eligible students in the selected classes were invited to participate. The original surveillance used predetermined sampling quotas by age, sex, and geographical stratum. Because the present study was a secondary analysis of an existing surveillance dataset, no separate *a priori* sample-size calculation was conducted for the mediation analysis, and all eligible records were considered.

The inclusion criteria were: (a) enrollment in Grades 1–6 at a primary school in Macao; (b) participation in the surveillance program; and (c) availability of data on extracurricular physical activity (EPA), screen time (ST), sleep duration (SD), and visual acuity (VA). Participants were excluded if they or their parent or guardian reported a congenital disease or disability that restricted participation in physical activity, or if variables required for the analysis contained missing or invalid values. The original questionnaires included a specific item assessing congenital diseases or disabilities that could restrict physical activity. The available dataset did not contain condition-specific diagnoses or severity criteria for organic ocular diseases, nor did it include a standardized cognitive assessment. Therefore, organic ocular disease and cognitive impairment were not treated as independently verified exclusion criteria in the present secondary analysis.

### 3.2 Data-collection procedures

Data were collected using standardized Chinese–Portuguese paper questionnaires and physical-health assessments administered at participating schools. The source of questionnaire responses differed by grade level. For students in Grades 1–3, the questionnaire included child-completed, parent-assisted, and parent-completed sections. The EPA and ST items were completed with parental or guardian assistance, whereas SD was reported in the parent-completed section. For students in Grades 4–6, EPA was primarily reported by the students, while parental or guardian assistance was permitted for the section containing the ST and SD items. Therefore, these measures were not considered exclusively child self-reported. Data were collected by trained surveillance personnel, including questionnaire administrators, physical-assessment examiners, medical personnel, and quality-control staff. The surveillance personnel were not involved in the participants’ academic grading. Standardized instructions were used, and completed forms were reviewed for missing or inconsistent responses. Macao is a highly urbanized region with variation in population density and access to recreational environments across residential areas. The questionnaire included selected items on digital-device access and environmental barriers to outdoor activity; however, comparable contextual measures were not available across all grades and were therefore not included in the primary mediation model.

### 3.3 Variable selection

#### 3.3.1 Extracurricular physical activity

EPA was assessed using two questionnaire items measuring weekly frequency and duration per session during the preceding month, excluding physical education classes. Weekly frequency ranged from no participation to seven or more sessions per week. The original reverse-ordered response codes were recoded as 0–7 sessions per week. Duration was categorized as less than 30 min, 30–59 min, 60–89 min, or 90 min or more and scored from 1 to 4. An ordinal EPA composite score was calculated by multiplying the recoded weekly frequency by the duration-category score. Participants reporting no EPA were assigned a score of 0. Higher scores represented greater EPA participation.

#### 3.3.2 Screen time

ST was assessed separately for school days and non-school days. Participants reported their usual daily time spent watching television or using smartphones, computers, or tablets. Responses were recorded using 10 ordered categories ranging from no screen use to 6 h or more per day. The no-screen-use category was recoded as 0, and the remaining categories retained ascending scores from 1 to 9. A weighted weekly ST score was calculated using five school days and two non-school days:

$\mathrm{ST}=\frac{5\times \text{school}-\mathrm{day}\;\text{score}+2\times \text{non}-\text{school}-\mathrm{day}\;\text{score}}{7}.$

Higher scores indicated longer total screen exposure. The questionnaire did not distinguish educational, recreational, or passive screen use.

#### 3.3.3 Sleep duration

SD, including daytime naps, was assessed separately for school days and non-school days. Responses were categorized as less than 8 h, 8 to less than 9 h, 9 to less than 10 h, 10 to less than 11 h, 11 to less than 12 h, or 12 h or more and scored from 1 to 6. A weighted weekly SD score was calculated using five school days and two non-school days. Higher scores indicated longer sleep duration. Sleep quality, sleep fragmentation, bedtime, and chronotype were not assessed.

#### 3.3.4 Visual acuity

Uncorrected distance VA was assessed separately for each eye at participating schools after questionnaire completion. A standard logarithmic visual-acuity chart was used at a distance of 5 m under standardized lighting conditions. Participants removed their spectacles before testing, and the right eye was assessed before the left eye. The original surveillance form recorded unaided VA, trial-lens correction results, categorical refractive status, and color-vision status for each eye. The exact clock time of the assessment was not retained in the analytical dataset. The poorer-eye unaided VA value was used in the analysis. Normal unaided VA was defined as a value of ≥5.0 in both eyes. Reduced unaided VA was defined as a value of <5.0 in either eye and categorized as mild (4.9), moderate (4.6–4.8), or severe (≤4.5). The term “reduced unaided visual acuity” was used rather than “visual impairment”, because the surveillance did not assess best-corrected VA or formally diagnose visual disability. The examination constituted school-based visual screening rather than a comprehensive ophthalmologic assessment. Consequently, the specific pathological cause of reduced VA could not be determined.

### 3.4 Statistical analysis

Statistical analyses were conducted using SPSS version 26.0 and AMOS version 24.0 (IBM Corp., Armonk, NY, USA). Descriptive statistics were used to summarize the demographic characteristics and core study variables. Pearson correlation analysis was conducted to assess the associations among the four core variables: extracurricular physical activity (EPA), screen time (ST), sleep duration (SD), and visual acuity (VA). Structural equation modeling was performed using AMOS 24.0 to examine the hypothesized direct and indirect associations among the variables. Model fit was evaluated using the chi-square-to-degrees-of-freedom ratio (χ^2^/df), comparative fit index (CFI), Tucker–Lewis index (TLI), root mean square error of approximation (RMSEA), and standardized root mean square residual (SRMR). The indirect effects were tested using bias-corrected bootstrap procedures with 5,000 resamples, and 95% confidence intervals (CIs) were calculated. An indirect effect was considered statistically significant when its 95% CI did not include zero. All statistical tests were two-sided, and *p* < 0.05 was considered statistically significant. Given the cross-sectional design, the estimated indirect effects were interpreted as statistical associations rather than evidence of temporal ordering or causal mechanisms.

## 4 Results

### 4.1 Demographics of study participants

A total of 2,249 primary-school records from Grades 1–6 were available in the surveillance dataset. Of these, 148 participants were excluded because they or their parent or guardian reported a congenital disease or disability that restricted participation in physical activity. One record was excluded because of missing bilateral visual-acuity data, one because of an unresolved invalid visual-acuity value, and one because of missing age information. The final analytical sample comprised 2,098 participants.

The participants ranged in age from 6 to 14 years, with a mean age of 8.36 ± 1.75 years. The sample included 1,223 boys (58.29%) and 875 girls (41.71%). Students from all six primary-school grades were represented. The distributions of age, sex, school grade, EPA, ST, sleep duration, and VA according to unaided-VA status are presented in Table 1. Compared with participants with normal unaided VA, those with reduced unaided VA were older (8.65 ± 1.76 vs. 7.99 ± 1.67 years, *p* < 0.001) and had higher ST scores (3.30 ± 1.96 vs. 2.95 ± 1.76, p < 0.001). The distribution of EPA also differed between the groups (*p* = 0.005), as did sex (*p* = 0.003) and grade distribution (*p* < 0.001). No statistically significant between-group difference was observed in the sleep-duration score (2.64 ± 0.83 vs. 2.66 ± 0.77, *p* = 0.436).

**Table 1:** Baseline characteristics of participants by unaided visual-acuity status.

Characteristic	Overall (*n* = 2,098)	Reduced VA (*n* = 1,191)	Normal VA (*n* = 907)	*p*-value
Age, years, mean ± standard deviation	8.36 ± 1.75	8.65 ± 1.76	7.99 ± 1.67	<0.001
Age range (minimum–maximum), years	6–14	6–14	6–13	–
Sex, *n* (%)				0.003
Boys	1,223 (58.29)	661 (55.50)	562 (61.96)	
Girls	875 (41.71)	530 (44.50)	345 (38.04)	
Grade, *n* (%)				<0.001
Grade 1	319 (15.20)	149 (12.51)	170 (18.74)	
Grade 2	381 (18.16)	168 (14.11)	213 (23.48)	
Grade 3	411 (19.59)	220 (18.47)	191 (21.06)	
Grade 4	347 (16.54)	226 (18.98)	121 (13.34)	
Grade 5	319 (15.20)	218 (18.30)	101 (11.14)	
Grade 6	321 (15.30)	210 (17.63)	111 (12.24)	
EPA ordinal index, median (IQR)	4.00 (2.00–8.00)	4.00 (2.00–6.00)	4.00 (2.00–8.00)	0.005
ST weighted ordinal score, mean ± standard deviation	3.15 ± 1.88	3.30 ± 1.96	2.95 ± 1.76	<0.001
Sleep duration weighted ordinal score, mean ± standard deviation	2.65 ± 0.80	2.64 ± 0.83	2.66 ± 0.77	0.436
Poorer-eye unaided VA, mean ± standard deviation	4.74 ± 0.35	4.52 ± 0.32	5.03 ± 0.05	–

Values are presented as mean ± standard deviation, median (IQR), or *n* (%), as appropriate. Reduced unaided VA was defined as unaided VA <5.0 in either eye, whereas normal unaided VA was defined as unaided VA ≥5.0 in both eyes. Between-group comparisons were performed using Welch’s independent-samples *t*-test for continuous variables, the Mann–Whitney *U* test for the EPA ordinal index, and Pearson’s chi-square test for categorical variables. No between-group statistical comparison was performed for poorer-eye unaided VA because VA status was defined using this measure. EPA, extracurricular physical activity; ST, screen time; VA, visual acuity; IQR, interquartile range.

### 4.2 Correlation analysis

The bivariate correlations among EPA, ST, SD, and VA are presented in Table 2. EPA was positively correlated with SD (*r* = 0.224, *p* < 0.01) and VA (*r* = 0.126, *p* < 0.01) and negatively correlated with ST (*r* = −0.051, *p* < 0.01). ST was negatively correlated with SD (*r* = −0.127, *p* < 0.01) and VA (*r* = −0.272, *p* < 0.01), whereas SD was positively correlated with VA (*r* = 0.253, *p* < 0.01).

**Table 2:** Pearson correlation coefficients (*n* = 2,098).

Variable	1	2	3	4
	*r*			
1. EPA	1			
2. ST	−0.051**	1		
3. SD	0.224**	−0.127**	1	
4. VA	0.126**	−0.272**	0.253**	1

^**^p < 0.01, based on two-tailed Pearson correlation tests. EPA, extracurricular physical activity; ST, screen time; SD, sleep duration; VA, visual acuity.

### 4.3 Model fit assessment

The results of the model fit assessment are listed in Table 3. The χ^2^/df value was 2.995 (<3). The root mean square error of approximation was 0.030 (<0.05). Regarding relative fit indices, the normed fit index (NFI = 0.987), incremental fit index (IFI = 0.991), Tucker–Lewis index (TLI = 0.985), and comparative fit index (CFI = 0.991).

**Table 3:** Model fit indices.

Fit Index	χ^2^/df	RMSEA	NFI	IFI	TLI	CFI
Criteria	<3	<0.08	>0.9	>0.9	>0.9	>0.9
Model fit results	2.995	0.030	0.987	0.991	0.985	0.991

RMSEA: root mean square error of approximation.

### 4.4 Path analysis

**Figure 2:**
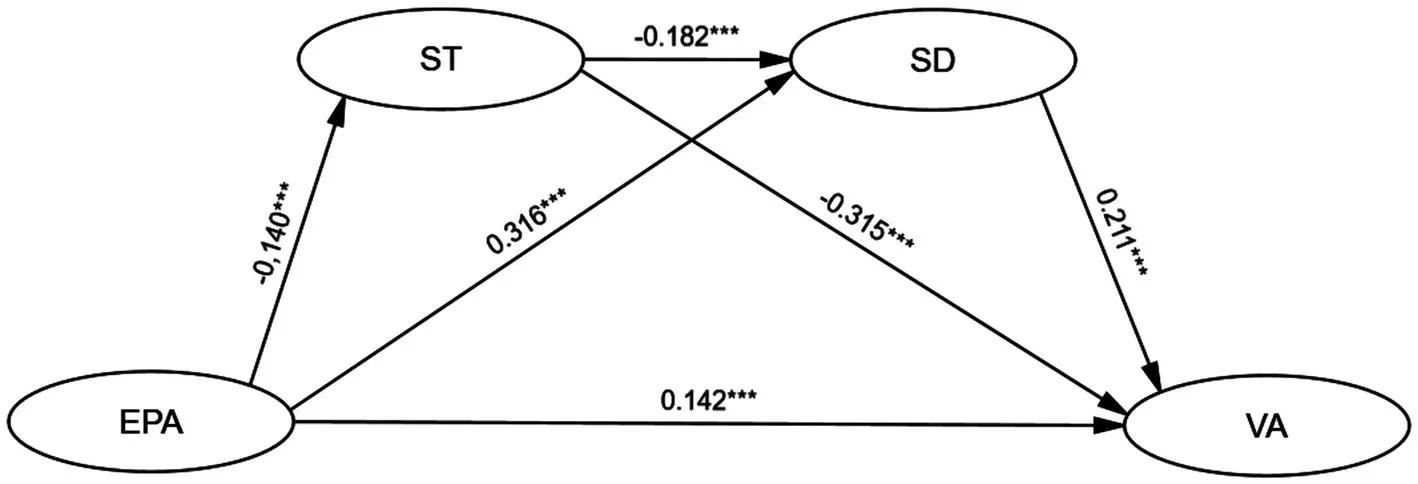
Path coefficient diagram. ^***^p < 0.001; EPA, extracurricular physical activity; ST, screen time; SD, sleep duration; VA, visual acuity.

The standardized path coefficients are presented in Table 4 and Figure 2. EPA was associated with VA (*β* = 0.142, *p* < 0.001), ST (*β* = −0.140, *p* < 0.001), and SD (*β* = 0.316, *p* < 0.001). ST was associated with SD (*β* = −0.182, *p* < 0.001) and VA (*β* = −0.315, *p* < 0.001), whereas SD was associated with VA (*β* = 0.211, *p* < 0.001).

**Table 4:** Path coefficient analysis among variables (*n* = 2,098).

Item	*β*	SE	*t*	*p*	95% CI	
					Lower	Upper
EPA → VA	0.142	0.015	4.605	0.000	0.113	0.171
EPA → ST	−0.140	0.037	−3.791	0.000	−0.338	−0.058
EPA → SD	0.316	0.044	7.402	0.000	0.230	0.402
ST → SD	−0.182	0.014	−4.910	0.000	−0.209	−0.155
ST → VA	−0.315	0.006	−9.492	0.000	−0.327	−0.303
SD → VA	0.211	0.015	6.522	0.000	0.182	0.240

EPA, extracurricular physical activity; ST, screen time; SD, sleep duration; VA, visual acuity.

### 4.5 Indirect-effect analysis

The direct, indirect, and total effects are presented in Table 5. The total effect of EPA on VA was statistically significant (*β* = 0.129, 95% CI [0.099, 0.162]). The direct effect was 0.071 (95% CI [0.038, 0.105]), accounting for 55.04% of the total effect. The total indirect effect through ST and SD was 0.058 (95% CI [0.039, 0.082]), accounting for 44.96% of the total effect. The specific indirect effect through ST was 0.022 (95% CI [0.010, 0.037]), representing 17.05% of the total effect, whereas the indirect effect through SD was 0.033 (95% CI [0.020, 0.052]), representing 25.58% of the total effect. The sequential indirect effect through ST and SD was 0.003 (95% CI [0.001, 0.006]), accounting for 2.33% of the total effect. None of the bootstrap confidence intervals included zero.

**Table 5:** Mediating effect and effect sizes (*n* = 2,098).

Path	*β*	Proportion of mediations in the total effect	95% CI	
			Lower	Upper
EPA → ST → VA	0.022	17.05%	0.010	0.037
EPA → SD → VA	0.033	25.58%	0.020	0.052
EPA → ST → SD → VA	0.003	2.33%	0.001	0.006
Total indirect effect	0.058	44.96%	0.039	0.082
Direct effect	0.071	55.04%	0.038	0.105
Total effect	0.129	100%	0.099	0.162

EPA, extracurricular physical activity; ST, screen time; SD, sleep duration; VA, visual acuity.

## 5 Discussion

### 5.1 Principal findings and descriptive context

This cross-sectional study examined the associations among extracurricular physical activity (EPA), screen time (ST), sleep duration (SD), and unaided visual acuity (VA) among primary school students in Macao. EPA was positively correlated with SD and VA and negatively correlated with ST. ST was negatively correlated with both SD and VA, whereas SD was positively correlated with VA. In the structural model, a direct association between EPA and VA remained after ST and SD were included, and non-zero indirect associations were observed through ST, SD, and the sequential ST–SD pathway. These findings were consistent with the hypothesized statistical model.

Reduced unaided VA, defined as a poorer-eye VA value below 5.0, was identified in 1,191 students (56.77%) in the analytical sample. This prevalence should be compared cautiously with that reported in comparable pediatric populations because estimates vary according to age, visual-acuity charts, screening thresholds, eye-specific classification, and whether unaided, presenting, or best-corrected VA is assessed. In addition, reduced unaided VA is a functional screening finding rather than a diagnosis of myopia or another specific ophthalmologic condition.

### 5.2 Bivariate associations among EPA, ST, SD, and VA

The positive correlation between EPA and VA was statistically significant but small in magnitude. Previous longitudinal and cross-sectional studies have reported associations of greater outdoor activity with slower axial elongation and lower risks of myopic shift among school-aged children (35). Exercise interventions conducted under outdoor illumination have also been associated with favorable visual or refractive outcomes (36), while systematic-review evidence suggests that outdoor physical activity may be relevant to the development of childhood myopia (37).

These studies provide a possible context for the present association, but they should not be interpreted as direct confirmation of the current VA findings. EPA and outdoor exposure are related but distinct constructs, and the present questionnaire did not determine whether the reported physical activity occurred indoors or outdoors. Moreover, the outcome of the present study was unaided VA rather than cycloplegic refractive error, axial length, or clinically diagnosed myopia.

Myopia is an important cause of reduced distance VA in school-aged children, but it is not the only possible explanation (38). Reduced unaided VA may also occur in association with other refractive abnormalities, particularly astigmatism, as well as amblyopia, strabismus, and other ocular conditions (39). Therefore, the association between EPA and VA cannot be interpreted as evidence that EPA prevented myopia or slowed myopic progression.

EPA was negatively correlated with ST, while ST was negatively correlated with VA. Participation in physical activity may coincide with less time allocated to sedentary screen-based behavior and prolonged near work, both of which have been linked to accommodative demand and visual fatigue (40). Previous studies have also reported that more physically active children tend to spend less time in sedentary screen-based activities (41, 42), and that prolonged screen exposure is associated with unfavorable refractive or visual outcomes in children (43, 44).

SD was positively correlated with both EPA and VA. Adequate sleep has been discussed as a restorative behavior potentially related to ocular recovery, visual performance, and circadian regulation (45). Previous studies have also reported associations of insufficient sleep with visual fatigue, altered accommodative function, or less favorable visual or refractive outcomes (46, 47). Regular physical activity may be associated with sleep through energy expenditure, daily routines, and circadian regulation (48). However, the present study assessed sleep duration only and did not measure sleep quality, regularity, fragmentation, bedtime, wake time, or chronotype. In addition, existing findings regarding sleep duration and refractive outcomes are not fully consistent. Therefore, the observed association between SD and VA should be interpreted cautiously and does not establish that longer sleep directly improves VA.

### 5.3 Individual and sequential mediating roles of ST and SD

The structural model suggested that the association between EPA and VA was not fully accounted for by ST and SD. Although both variables contributed to the estimated indirect association, a direct association between EPA and VA remained after they were included in the model. This pattern indicates that ST and SD may represent only part of the behavioral context linking EPA with VA. Other characteristics accompanying physical activity, such as outdoor exposure, reduced near-work time, daily routine, or broader family and school environments, may also be relevant. However, these factors were not directly measured in the present study and should be regarded as possible explanations rather than confirmed mechanisms.

The indirect pathway through ST is consistent with a time-displacement perspective. Children have a limited amount of discretionary time, and greater participation in EPA may coincide with less time available for television viewing and the use of smartphones, computers, or tablets. Previous research has similarly emphasized that physical activity and screen-based sedentary behavior compete for time within children’s daily routines (20, 49). Reduced screen exposure may also correspond to less prolonged near viewing and fewer uninterrupted periods of visually demanding activity. Nevertheless, the present study combined several types of screen use and did not distinguish educational from recreational exposure. The indirect association through ST therefore cannot be attributed to a particular device, viewing distance, or screen-use purpose.

SD also contributed to the association between EPA and VA. Regular physical activity has been associated with sleep through energy expenditure, daily scheduling, and circadian regulation (31, 44). At the same time, children with more consistent sleep routines may have better-regulated daily behaviors, including greater participation in physical activity and lower screen exposure. The observed SD pathway may therefore reflect not only sleep duration itself, but also a broader pattern of organized daily routines.

The sequential EPA–ST–SD–VA pathway was statistically detectable but accounted for only a small proportion of the total association. This modest estimate suggests that the proposed sequence is unlikely to represent a dominant pathway. Rather, ST and SD may operate as interrelated components of a broader daily time-use system in which physical activity, sedentary behavior, and sleep mutually constrain one another (50). The cross-sectional design did not establish whether greater EPA preceded lower ST and longer SD, or whether children with different sleep and screen-use patterns were more likely to participate in EPA.

### 5.4 Bidirectional and reverse associations

Bidirectional associations should also be considered. Children with reduced VA may encounter difficulties in activities requiring distance judgment, rapid visual tracking, ball control, or navigation in outdoor environments and may therefore participate less frequently in some forms of physical activity. Previous research found that adolescents with severe visual impairments engaged in less physical activity than those without visual impairments, supporting the plausibility that visual limitations may influence subsequent activity participation (51). However, severe visual impairment differs from the reduced unaided VA assessed in the present study, and this evidence should therefore be interpreted cautiously. Children with reduced VA might also be more likely to engage in indoor or close-view activities, although this possibility was not directly examined in the present study.

Conversely, ST, SD, and physical activity may influence one another over time. A longitudinal study of Australian children identified bidirectional associations between screen time and sleep duration from 10 to 12 years of age and also examined the role of physical activity in these relationships (52). Sleep duration may influence daytime activity and screen-use patterns, while physical activity and screen exposure may subsequently affect sleep. The observed paths may therefore represent a dynamic and interdependent behavioral system rather than a single unidirectional sequence from EPA to ST, SD, and VA.

### 5.5 Theoretical and practical contributions

The present study extends previous research by examining EPA, ST, SD, and unaided VA within a single statistical model. The findings are consistent with the view that children’s daily behaviors are interrelated and that the association between EPA and VA may coexist with differences in screen use and sleep duration.

From a practical perspective, schools and families may adopt an integrated approach that provides regular opportunities for physical activity, encourages appropriate breaks during prolonged screen use, supports consistent sleep routines, and strengthens routine visual-acuity screening. Outdoor activity may be encouraged where conditions are safe and suitable.

### 5.6 Limitations and future directions

The cross-sectional design did not establish temporal ordering among EPA, ST, SD, and VA. Accordingly, the estimated indirect effects should not be interpreted as evidence of causal mediation, and reverse or bidirectional relationships remain possible. EPA, ST, and SD were measured using ordered questionnaire categories, with responses provided by children, completed with parental or guardian assistance, or reported by parents or guardians depending on grade and questionnaire section. Recall error, social-desirability bias, differences between self- and proxy-reporting, and exposure misclassification may therefore have affected the behavioral measures. EPA did not distinguish indoor from outdoor activity or objectively quantify activity intensity. ST did not differentiate device type, educational and recreational use, viewing distance, or continuous exposure, whereas SD did not capture sleep quality, regularity, fragmentation, bedtime, or chronotype.

Unaided VA was assessed through school-based screening rather than comprehensive ophthalmologic examination. Cycloplegic refraction, spherical equivalent, axial length, and other clinical diagnostic information were unavailable; therefore, the causes of reduced VA could not be determined, and the findings cannot be interpreted specifically as associations with myopia. Participants reporting congenital diseases or disabilities that restricted physical activity were excluded on the basis of a single questionnaire item. However, mild, acquired, unreported, or undiagnosed physical limitations may have remained and may have influenced both EPA and VA. Residual confounding also cannot be excluded because several potentially relevant factors were not measured consistently across all grades, including parental refractive status, household socioeconomic conditions, access to electronic devices, outdoor light exposure, homework and near-work demands, access to recreational facilities, neighborhood safety, traffic, population density, and residential environment.

The study population was limited to primary school students in Macao, and the findings may not be generalizable to populations with different educational, behavioral, socioeconomic, or residential contexts. Future longitudinal research should use repeated assessments to clarify temporal relationships and incorporate accelerometry, objective screen-use tracking, actigraphy, outdoor-light sensors, and comprehensive ophthalmologic examinations. Multilevel analyses that account for family, school, and neighborhood characteristics may further clarify the individual and contextual factors underlying the observed associations. Despite these limitations, the present study provides a basis for further investigation of the interrelationships among EPA, ST, SD, and unaided VA in school-aged children.

## 6 Conclusion

This study identified a positive association between EPA and unaided VA among primary school students. Higher EPA was also associated with lower ST and longer SD, and statistically detectable indirect associations with VA were observed through ST, SD, and the sequential ST–SD pathway. These findings indicate that EPA, ST, SD, and unaided VA are interrelated within children’s daily behavioral patterns. However, the cross-sectional design does not establish temporal ordering or causal mediation. School and public health strategies may therefore consider opportunities for physical activity, appropriate management of prolonged screen use, consistent sleep routines, and routine visual-acuity screening as complementary components of child health promotion.

## Data Availability

The raw data supporting the conclusions of this article will be made available by the authors, without undue reservation.
