# Supplementary material for: The mediating roles of screen time and sleep duration in the association between extracurricular physical activity and visual acuity in primary school students
Source: Front Public Health. 2026 Sep 3;14:1883751. doi: 10.3389/fpubh.2026.1883751 (PMC13582207; doi:10.3389/fpubh.2026.1883751)
Supplement: Supplementary file 1 [file Data_Sheet_1.pdf]

主辦機構：
Entidade Organizadora:

澳門特別行政區政府體育局
Instituto do Desporto do Governo da RAEM

澳門羅理基博士大馬路818號
Av. Dr. Rodrigo Rodrigues, nº. 818, Macau

www.sport.gov.mo

技術支援：
Apoio Técnico:

國家體育總局體育科學研究所
Instituto de Investigação das Ciências Desportivas da Administração Geral do Desporto da China

協辦機構：
Entidades Colaboradoras:

衛生局
Serviços de Saúde

教育暨青年局
Direcção dos Serviços de Educação e Juventude

澳門特別行政區政府社會工作局
GOVERNO DA RAEM INSTITUTO DE ACÇÃO SOCIAL

澳門特別行政區政府
Governo da Região Administrativa Especial de Macau
高等教育局
Direcção dos Serviços do Ensino Superior

澳門理工學院
Instituto Politécnico de Macau

2020 F

澳門市民體質監測

Avaliação da Condição Física da População de Macau

數據登錄冊
Registo de Dados

兒童青少年(學生：6~22歲)
Crianças e adolescentes (estudantes dos 6 aos 22 anos)

(小學四年級～小學六年級學生)
(Alunos do 4 º Ano ao 6 º Ano do ensino primário)

澳門特別行政區政府體育局
Instituto do Desporto do Governo da RAEM

歡迎　閣下參加2020年澳門市民體質監測！澳門市民體質監測是特區政府為推動大眾健身活動的開展而進行的調查研究。在此首先感謝　閣下對監測的支持並認真、如實地填寫問卷，我們保證對　閣下的個人資料保密，有關資料不會獨立出現或使用，它將是整體數據的組成部分。再次對　閣下的真誠參與表示感謝！

如對調查內容和檢測項目有任何疑問，歡迎向體育局運動醫學中心垂詢！

電話：28810896，88934540

Bem-vindo à Avaliação da Condição Física da População de Macau 2020! Este é um inquérito promovido pelo Governo da RAEM com vista a desenvolver as actividades do desporto para todos. Agradecemos que preencha o presente inquérito com o maior rigor possível. Garantimos que todas as suas informações pessoais serão mantidas em total confidencialidade e que, sendo parte integrante do conjunto de dados, nunca serão reveladas ou utilizadas independentemente. Mais uma vez, agradecemos muito a sua disponibilidade e colaboração!

Em caso de dúvidas sobre o conteúdo do inquérito ou dos testes, é favor contactar o Centro de Medicina Desportiva do Instituto do Desporto pelos telefones 28810896 e 88934540.

體育局轄下運動醫學中心預先聲明
Centro de Medicina Desportiva do Instituto do Desporto
Declaração prévia

本聲明旨在向個人資料當事人或相關家長或監護人確保遵守第8/2005號法律“個人資料保護法”所規範的事宜。

A presente declaração visa assegurar ao titular dos dados pessoais ou ao respectivo encarregado de educação ou tutor o respeito pelo disposto na Lei n.º 8/2005 (Lei de Protecção de Dados Pessoais)

1. 處理個人資料的負責人—體育局，地址設於澳門羅理基博士大馬路818號。代表—體育局局長。

   Responsável pelo tratamento dos dados pessoais - Instituto do Desporto, Av. Dr. Rodrigo Rodrigues, n.º 818, Macau. Representante - Presidente do Instituto do Desporto.

2. 處理個人資料的目的—進行澳門市民體質監測，以便為制定日後關於體育及醫療護理的政策提供科學數據。收集的數據旨在更新僅為統計用途的數據庫。

   Finalidades do tratamento dos dados pessoais - Avaliação da condição física da população da RAEM com vista à obtenção de dados de natureza científica que sirvam de base à definição das futuras linhas políticas em matéria de desporto e cuidados de saúde. Os dados recolhidos destinam-se à actualização de base de dados para fins estatísticos, não podendo ser utilizados ou revelados para outros fins.

3. 個人資料當事人類別 — 參與是次計劃的澳門居民（隨年齡抽樣）。

   Categorias de titulares dos dados pessoais - A população da RAEM que participe neste projecto (por amostragem etária).

4. 個人資料接收者—個人資料當事人（或相關家長或監護人），運動醫學中心及國家體育總局體育科學研究所。

   Destinatários dos dados pessoais - Titular dos dados pessoais (ou o respectivo encarregado de educação ou tutor), Centro de Medicina Desportiva e Instituto de Investigação das Ciências Desportivas da Administração Geral do Desporto da China.

5. 接收及處理個人資料的條件—個人資料當事人（或相關家長或監護人）享有第8/2005號法律賦予的所有權利如查閱權及更正所收集的個人資料。運動醫學中心承諾對不正確的數據進行更正，刪除或封鎖。

   Condições de recolha e tratamento dos dados pessoais - O titular dos dados pessoais (ou o respectivo encarregado de educação ou tutor) goza de todos os direitos que lhe são conferidos pela Lei n.º 8/2005 (Lei de Protecção de Dados Pessoais), designadamente do direito de acesso e de rectificação dos dados pessoais recolhidos. O Centro de Medicina Desportiva compromete-se a rectificar, a apagar ou a bloquear os dados inexactos.

6. 處理個人資料的安全和保密性—對處理及編輯個人資料絕對保密及採取適當措施以確保其安全。

   Segurança dos dados e confidencialidade no tratamento dos dados pessoais - o tratamento e o processamento de dados estão sujeitos a total confidencialidade e ao respeito de medidas idóneas a garantir a respectiva segurança.

本人已明白上述內容權益
Tomei conhecimento

____

（個人資料當事人或家長或監護人簽名）
(Assinatura do titular dos dados ou do respectivo encarregado de educação ou tutor)

_______ / _______ / _______
（日 Dia）（月 Mês）（年 Ano）

姓　　名：
Nome: ______________________

性　　別：
Sexo: ______________________

年　　齡：　　　　　　（周歲）
Idade: ______________________ (Anos)

就讀學校：
Escola: ______________________

聯繫電話：
Telefone: ______________________

一.分類編碼（學生個人資料，小學生可由家長協助填寫）

Codificação(Dados pessoais do aluno. Os alunos do ensino primário podem pedir auxílio ao encarregado de educação para o preenchimento)

填寫方法：

填寫選擇題時請將選擇答案的序號填入相應的方格中，如選答案（1），則在方格內填1；序號為兩位數時，兩位數字填入同一方格，如選項為（11），則將 [11] 填入同一方格，即填為 [11]。在填寫多項選擇題時，如果僅選擇一個或兩個答案，則應在剩餘方格內填0。

Instruções para o preenchimento do questionário:

Preencha o número escolhido representativo da sua resposta no quadrado correspondente, como, por exemplo, se escolher a resposta (1), deve escrever “1” no quadrado fornecido. Quando o número representativo da resposta tiver dois algarismos, estes devem ser preenchidos no mesmo quadrado, por exemplo, para a opção número (11), o “[11]” deve aparecer no mesmo quadrado, ou seja, [11]. E, no caso de múltiplas opções e quando só parte das opções forem escolhidas, deverá preencher “0” nos restantes quadrados.

1.澳門居民身份證號碼
Número do BIR da RAEM
□□□□□□□□

2.性別 Sexo
(1) 男 Masculino
(2) 女 Feminino
□

3.出生日期
Data de nascimento
□□□□ 年 Ano □□ 月 Mês □□ 日 Dia

4.測試日期（由監測人員填寫）
Data da avaliação *(a preencher pelo avaliador)*
□□□□ 年 Ano □□ 月 Mês □□ 日 Dia

5.學校代碼（由監測人員填寫）
Código da escola *(a preencher pelo avaliador)*
□□□□

6.測試序號（由監測人員填寫）
Numeração do avaliado *(a preencher pelo avaliador)*
□□□□

7.居住在澳門的年限（年）(指連續在澳門居住的年限，如果離開澳門的時間超過1年，則要由回澳門居住起重新計算居澳年限)
Tempo de residência em Macau *(em anos). (Refere-se ao número ininterrupto de anos de residência em Macau. Caso tenha estado ausente de Macau por um período superior a 1 ano, o período de residência em Macau é calculado a partir da data do regresso)*
□□

8.在學校就讀年級
Grau de ensino que frequenta
(1) 小學四年級 4º Ano do ensino primário
(2) 小學五年級 5º Ano do ensino primário
(3) 小學六年級 6º Ano do ensino primário
□

9.出生地
Naturalidade
(1) 中國大陸 China
(2) 澳門 Macau
(3) 香港 Hong Kong
(4) 葡萄牙 Portugal
(5) 其他 Outro
□

10.居住地所屬堂區
Paróquia onde reside
□

(1)聖方濟各堂區（路環）
Paróquia de São Francisco Xavier *(Coloane)*

(2)聖嘉模堂區（氹仔）
Paróquia de Nossa Senhora do Carmo *(Taipa)*

(3)風順堂區（包括媽閣山、西望洋山、下環及內港一帶）
Paróquia de S. Lourenço *(Zonas das Colinas da Barra e da Penha, da Praia do Manduco e do Porto Interior)*

(4)大堂區（新馬路一帶、南灣、水坑尾、新口岸南北地段及南灣湖發展區）
Paróquia da Sé Catedral *(Zonas da Almeida Ribeiro, da Praia Grande, da Rua do Campo, dos Lotes norte e sul do Porto Exterior e da Zona do Lago Nam Van)*

(5)花王堂區（聖安多尼堂區，在澳門西部，包括高士德大馬路、新橋和沙梨頭）
Paróquia de Santo António *(Zonas da Freguesia de Santo António, do Oeste de Macau, incluindo Av. Horta e Costa, de San Kiu e do Patane)*

(6)望德堂區（包括荷蘭園、松山一帶）
Paróquia de S. Lázaro *(Zona do Conselheiro Ferreira de Almeida e da Colina da Guia)*

(7)花地瑪堂區（俗稱北區，包括青洲、台山、黑沙環、筷子基和水塘)
Paróquia de Nossa Senhora de Fátima *(Zona Norte, incluindo Ilha Verde, Tamagnini Barbosa, Areia Preta, Fai Chi Kei e Reservatório)*

11.近5年曾患過何種疾病（經醫院確診的疾病）（選擇“無”者請直接回答問題第13題）
Sofreu de algumas doenças nos últimos 5 anos *(com registo médico)? (Se a resposta for negativa, passe directamente para a pergunta 13)*
□

(1) 有 Sim
(2) 無 Não

12.患病種類（患者者按主次順序，最多可填寫3項）
Tipo de doenças sofridas *(Pode indicar no máximo 3, por ordem de gravidade)*

(1) 氣管炎 Traqueíte	(2) 肺炎 Pneumonia	(3) 哮喘 Asma
(4) 過敏性鼻炎 Rinite alérgica	(5) 腹瀉病 Doença diarreica	(6) 腸胃炎 Gastroenterite
(7) 腎炎 Nefrite	(8) 扁桃腺炎 Amigdalite	(9) 肝炎 Hepatite
(10) 肺結核 Tuberculose	(11) 水痘 Varicela	(12) 脊柱彎曲異常 Escoliose
(13) 流行性腮腺炎 Doença da mão, pé e boca	(14) 貧血 Anemia	(15) 心臟病 Doenças cardíacas
(16) 糖尿病 Diabetes	(17) 意外傷害(造成人體的損傷而需要醫治或影響了正常的活動) Traumatismo por acidente *(Lesões físicas que obrigaram o internamento ou que afectaram a sua actividade regular)*	(18) 其他 Outros

13.有沒有每天刷牙習慣
Tem hábitos de escovar diariamente os dentes?

(1) 有 Sim　(2) 無 Não

14.除刷牙外，有沒有每天使用牙線
Se sim, além de escovar os dentes, usa diariamente fio dental?

(1) 有 Sim　(2) 無 Não

15.過去12個月有沒有到牙醫診所進行牙科檢查
Nos últimos 12 meses, foi a clínicas dentárias para a realização de exames dentários?

(1) 有 Sim　(2) 無 Não

16.是否有齲齒（選擇“無”或“不知道”者請直接回答問題第18題）
Tem dentes cariados? *(Se a resposta for negativa ou não souber, passe directamente para a pergunta 18)*

(1) 有 Sim　(2) 無 Não　(3) 不知道 Não sei

17.如有齲齒，有沒有到牙醫診所進行治療?
Se tiver dentes cariados, foi a clínicas dentárias para o tratamento?

(1) 有 Sim　(2) 無 Não

18.是否有親兄弟姐妹?
Tem irmãos?

(1) 沒有 Não　(2) 有1個 1　(3) 有2個或以上 2 ou mais

19.是否有影響開展體育活動的先天性疾病或者殘疾?
Tem doenças ou deficiências congénitas que o/a impedem praticar actividades físicas?

(1) 沒有 Não　(2) 有 Sim（請注明 Especifique ______________）

20.是否與父母住在一起共同生活?
Vive juntamente com os pais?

(1) 是 Sim　(2) 否 Não　(3) 只與父或母任一方同住 Apenas com uma das partes – Pai ou Mãe

二.健身素養問卷 Questionário de aptidão física

A-健身知識(由學生獨立回答)
Conhecimentos sobre a prática de exercício físico *(A preencher pelo aluno)*

A1.【單選題】您認為以下說法是否正確，正確選1、不正確選2、不知道選3，並在您選的數字上畫“〇”。

【Perguntas de escolha múltipla (com resposta única)】Acha que as seguintes afirmações estão correctas? Se estiver correcta, escolha 1; se estiver incorrecta, escolha 2; se não souber a resposta, escolha 3 e assinale um círculo “〇” à volta do número correspondente.

題目<br>Tema	正確<br>Correcta	不正確<br>Incorrecta	不知道<br>Não sei
1) 劇烈運動時，只能用鼻子呼吸<br>Quando pratica exercício físico intenso, apenas se pode respirar pelo nariz	1	2	3
2) 運動時抽筋，應該立即休息<br>Quando ocorrem cãibras durante a prática de exercício físico, deve-se descansar de imediato	1	2	3
3) 每次鍛煉前要做必要的伸展運動<br>Antes da prática de exercício físico, deve-se realizar exercícios de alongamento	1	2	3
4) 彎道跑時，身體要向跑道內傾斜<br>Durante a corrida, ao aproximar-se das curvas, o corpo deve inclinar-se para o interior da pista	1	2	3
5) 體育鍛煉後可以立即吃飯<br>Pode-se comer logo após a prática de exercício físico	1	2	3
6) 運動前不能吃太飽<br>Não se deve comer muito antes da prática de desporto	1	2	3
7) 運動後，為緩解疲勞，可以多吃一些蔬菜和水果<br>Depois da prática de exercício físico, pode-se comer mais legumes e frutas de modo a aliviar a fadiga	1	2	3
8) 蹲踞式起跑需要在聽到“預備”口令後，抬起臀部，身體重心同時前移，形成臀部高於肩、肩超過起跑線的身體姿勢<br>A técnica de “saída baixa” refere-se a uma forma de iniciar a corrida com apoio do bloco de partida, sendo composto por três fases, nomeadamente “aos seus lugares”, “pronto “e “corrida”. Ao ouvir “pronto”, o atleta aproxima-se ao bloco de partida e coloca os seus pés sobre os suportes dos blocos de partida	1	2	3
9) 站立體前屈需要兩腿並立，膝蓋伸直，上體與腿盡量貼近<br>NaA flexão ventral de pé, as pernas devem ser colocadas lado a lado e os joelhos esticados, por forma a aproximar o tronco o mais perto possível das pernas	1	2	3
10) 原地高抬腿運動的節奏要快，身體需要微微向後傾<br>O ritmo do exercício de levantamento da perna no local deve ser rápido, inclinando o tronco ligeiramente para trás	1	2	3

B-健身態度(由學生獨立回答)
Atitude da prática do exercício físico *(A preencher pelo aluno)*

B1.【單選題】以下說法你是否同意，非常不同意選1、不太同意選2、一般選3、比較同意選4、非常同意選5，並在你選的數字上畫“〇”

【Perguntas de escolha múltipla (com resposta única)】Concorda com as seguintes afirmações? Se não concordar totalmente, escolha 1; se não concordar parcialmente, escolha 2; se for indiferente, escolha 3; se concordar parcialmente, escolha 4; se concordar totalmente, escolha 5 e assinale um círculo “〇” à volta do número correspondente.

題目<br>Tema	非常不同意<br>Não concordo totalmente	不太同意<br>Não concordo parcialmente	一般<br>Indiferente	比較同意<br>Concordo parcialmente	非常同意<br>Concordo totalmente
1) 體育鍛煉可以強身健體<br>A prática de exercício físico pode reforçar a condição física	1	2	3	4	5
2) 體育鍛煉有利於減少脂肪，避免過度肥胖<br>O exercício físico ajuda a reduzir a gordura corporal e a evitar a obesidade excessiva	1	2	3	4	5
3) 體育鍛煉有助於增強自信心<br>O exercício físico pode ajudar a aumentar a autoconfiança	1	2	3	4	5
4) 體育鍛煉能夠讓自己交到更多朋友<br>A prática de exercício físico pode ajudar a fazer novas amizades	1	2	3	4	5

B2.【單選題】以下說法是否符合你的真實情況?非常不符合選1、不太符合選2、一般選3、比較符合選4、非常符合選5，並在你選的數字上畫“〇”。

【Perguntas de escolha múltipla (com resposta única)】Assinale as escolhas de acordo com o seu ponto de vista real. Se não corresponder totalmente, escolha 1; se não corresponder parcialmente, escolha 2; se for indiferente, escolha 3; se corresponder parcialmente, escolha 4; se corresponder totalmente, escolha 5 e assinale um círculo “〇” à volta do número correspondente.

題目<br>Tema	非常不符合<br>Não corresponde totalmente	不太符合<br>Não corresponde parcialmente	一般<br>Indiferente	比較符合<br>Corresponde parcialmente	非常符合<br>Corresponde totalmente
5) 體育鍛煉活動非常有用<br>A prática de exercício físico é bastante útil	1	2	3	4	5
6) 體育鍛煉活動非常無聊<br>A prática de exercício físico é uma actividade bastante aborrecida	1	2	3	4	5
7) 體育鍛煉活動對身體有害<br>A prática de exercício físico é prejudicial ao corpo	1	2	3	4	5
8) 體育鍛煉的體驗不是很愉快<br>A experiência obtida na prática de exercício físico não foi muito agradável	1	2	3	4	5

B3.【單選題】以下説法是否符合你的真實情況?非常不符合選1、不太符合選2、一般選3、比較符合選4、非常符合選5，並在你選的數字上畫“〇”。

【Perguntas de escolha múltipla (com resposta única)】Assinale as escolhas de acordo com o seu ponto de vista real. Se não corresponder totalmente, escolha 1; se não corresponder parcialmente, escolha 2; se for indiferente, escolha 3; se corresponder parcialmente, escolha 4; se corresponder totalmente, escolha 5 e assinale um círculo “〇” à volta do número correspondente.

題目<br>Tema	非常不符合<br>Não corresponde totalmente	不太符合<br>Não corresponde parcialmente	一般<br>Indiferente	比較符合<br>Corresponde parcialmente	非常符合<br>Corresponde totalmente
9) 完成體育課的要求非常困難<br>É muito difícil concluir os requisitos das aulas de educação física	1	2	3	4	5
10) 父母或對我很重要的人（比如爺爺、奶奶、同學、朋友等）不會要求我進行體育鍛煉<br>Os pais ou pessoas que são importantes para mim (como avô, avó, colegas de escola ou amigos), não me exigem a praticar exercício físico	1	2	3	4	5
11) 如果我願意，體育鍛煉要求的各項指標我都可以達標<br>Se eu estiver disposto, posso satisfazer todos os indicadores necessários à prática de exercício físico	1	2	3	4	5

B4.【單選題】以下説法是否符合你的真實情況?非常不符合選1、不太符合選2、一般選3、比較符合選4、非常符合選5，並在你選的數字上畫“〇”。

【Perguntas de escolha múltipla (com resposta única)】Assinale as escolhas de acordo com o seu ponto de vista real. Se não corresponder totalmente, escolha 1; se não corresponder parcialmente, escolha 2; se for indiferente, escolha 3; se corresponder parcialmente, escolha 4; se corresponder totalmente, escolha 5 e assinale um círculo “〇” à volta do número correspondente.

題目<br>Tema	非常不符合<br>Não corresponde totalmente	不太符合<br>Não corresponde parcialmente	一般<br>Indiferente	比較符合<br>Corresponde parcialmente	非常符合<br>Corresponde totalmente
12) 看到有同學或朋友參加體育鍛煉，就想參與其中<br>Quando os colegas ou amigos praticarem exercício físico também quer participar	1	2	3	4	5
13) 能做到一直堅持參加體育鍛煉<br>É capaz de praticar de forma contínua o exercício físico	1	2	3	4	5
14) 給自己制定了一份體育鍛煉的計劃<br>Elaborou um plano de exercício físico formulado para si próprio	1	2	3	4	5

C-健身技能(由學生獨立回答) Habilidades físicas *(a preencher pelo aluno)*

C1.【多選題】你嘗試過的運動項目有哪些？本題爲多選題，可選1個或1個以上的答案，請圈選所有你參與過的運動項目，在選項對應的序號上畫“〇”。

【Perguntas de escolha múltipla (com resposta múltipla)】Quais foram as modalidades desportivas que já praticou？Esta parte é constituída por perguntas de escolha múltipla, podendo assinalar uma ou mais respostas para indicar todas as modalidades que praticou. Assinale um círculo “〇” à volta do número correspondente.

1	健步走<br>Caminhada	13	騎自行車<br>Ciclismo	25	水球<br>Pólo aquático
2	中短程跑(1500米內)<br>Corrida de meio-fundo *(1500 metros)*	14	羽毛球<br>Badminton	26	柔道<br>Judo
3	長跑(距離超過1500米)<br>Corrida de fundo *(distância superior a 1500 metros)*	15	網球<br>Ténis	27	滑冰<br>Patinagem no gelo
4	跳高<br>Salto em altura	16	體操<br>Ginástica	28	滑雪<br>Esqui
5	跳遠<br>Salto em comprimento	17	韻律操（形體操、健美操、體育舞蹈、舞蹈等）<br>Ginástica rítmica *(aeróbica, ginástica, dança desportiva, dança etc.)*	29	跳繩<br>Salto à corda
6	籃球<br>Basquetebol	18	游泳<br>Natação	30	踢毽子<br>Shuttlecock *(jogo da peteca)*
7	排球(包括沙灘排球)<br>Voleibol *(Incluindo voleibol de praia)*	19	武術<br>Wushu	31	輪滑<br>Patinagem
8	足球<br>Futebol	20	跆拳道<br>Taekwondo	32	冰球<br>Hóquei no gelo
9	乒乓球<br>Ténis-de-mesa	21	空手道<br>Karate-do	33	舉重<br>Halterofilismo
10	高爾夫球<br>Golfe	22	曲棍球<br>Hóquei	34	標槍<br>Lançamento do dardo
11	棒球<br>Basebol	23	擊劍<br>Esgrima	35	其他<br>Outros
12	鉛球(包括實心球)<br>Lançamento de peso	24	跨欄<br>Corrida com barreiras	36	沒有參加過任何運動項目(不用回答C2題)<br>Não pratiquei nenhuma modalidade desportiva *(Não necessita responder à pergunta C2)*

C2.【多選題】你能夠熟練掌握的運動項目有哪些（熟練是指自己能掌握某項運動的基本技能）？本題爲多選題，可選1個或1個以上的答案，請圈選所有你熟練掌握的運動項目，在選項對應的序號上畫“○”。

【Perguntas de escolha múltipla (com resposta múltipla)】Quais são as modalidades desportivas que domina com maior proficiência? (ter proficiência significa dominar, por si próprio, as habilidades básicas de um desporto). Esta parte é constituída por perguntas de escolha múltipla, podendo assinalar uma ou mais respostas para indicar as modalidades que domina com maior proficiência . Assinale um círculo “○” à volta do número correspondente.

1	健步走<br>Caminhada	13	騎自行車<br>Ciclismo	25	水球<br>Pólo aquático
2	中短程跑(1500米內)<br>Corrida de meio-fundo *(1500 metros)*	14	羽毛球<br>Badminton	26	柔道<br>Judo
3	長跑(距離超過1500米)<br>Corrida de fundo *(distância superior a 1500 metros)*	15	網球<br>Ténis	27	滑冰<br>Patinagem no gelo
4	跳高<br>Salto em altura	16	體操<br>Ginástica	28	滑雪<br>Esqui
5	跳遠<br>Salto em comprimento	17	韻律操（形體操、健美操、體育舞蹈、舞蹈等）<br>Ginástica rítmica *(aeróbica, ginástica, dança desportiva, dança etc.)*	29	跳繩<br>Salto à corda
6	籃球<br>Basquetebol	18	游泳<br>Natação	30	踢毽子<br>Shuttlecock *(jogo da peteca)*
7	排球(包括沙灘排球)<br>Voleibol *(Incluindo voleibol de praia)*	19	武術<br>Wushu	31	輪滑<br>Patinagem
8	足球<br>Futebol	20	跆拳道<br>Taekwondo	32	冰球<br>Hóquei no gelo
9	乒乓球<br>Ténis-de-mesa	21	空手道<br>Karate-do	33	舉重<br>Halterofilismo
10	高爾夫球<br>Golfe	22	曲棍球<br>Hóquei	34	標槍<br>Lançamento do dardo
11	棒球<br>Basebol	23	擊劍<br>Esgrima	35	其他<br>Outros
12	鉛球(包括實心球)<br>Lançamento de peso	24	跨欄<br>Corrida com barreiras	36	沒有熟練掌握的運動項目<br>Não domino nenhuma modalidade desportiva com proficiência

C3.【多選題】你能夠熟練掌握的準備活動有哪些（熟練是指自己獨立會做某項準備活動）？本題爲多選題，可選1個或1個以上的答案，請圈選所有你熟練掌握的準備活動，在選項對應的序號上畫“○”。如果沒有熟練掌握的準備活動只圈選10即可。

【Perguntas de escolha múltipla (com resposta múltipla)】Quais são os exercícios de preparação que domina com maior proficiência? (ter proficiência significa conseguir fazer esse exercício por si próprio). Esta parte é constituída por perguntas de escolha múltipla, podendo assinalar uma ou mais respostas para indicar os exercícios de preparação que domina com maior proficiência. Assinale um círculo “○” à volta do número correspondente e se não domina nenhum exercício de preparação com proficiência, basta assinalar o número 10.

1	慢跑<br>Jogging	6	壓腿（包括前壓腿和側壓腿）<br>Pressão de pernas *(leg press)* *(incluindo movimentos da parte frontal e lateral)*
2	腰部運動（如扭腰）<br>Movimento da cintura *(por exemplo, rotação da cintura)*	7	體前屈<br>Flexão ventral
3	肩部運動（轉動肩膀）<br>Movimento de ombros *(movimento giratórios dos ombros)*	8	原地高抬腿<br>Perna erguida no local
4	關節活動（包括手腕關節、頸關節和腳踝關節）<br>Movimento das articulações *(incluindo articulações dos punhos, do pescoço e dos tornozelos)*	9	其他<br>Outros
5	擴胸運動<br>Exercícios de expansão torácica	10	沒有熟練掌握的準備活動<br>Não domino nenhum exercício de preparação com proficiência

C4.【多選題】你知道以下哪些運動損傷的正確處理方法？本題爲多選題，可選1個或1個以上的答案，請圈選所有你會處理的運動損傷情況，在選項對應的序號上畫“○”。

【Perguntas de escolha múltipla (com resposta múltipla)】Quais são os tratamentos mais correctos a adoptar para as seguintes lesões desportivas? Esta parte é constituída por perguntas de escolha múltipla, podendo assinalar uma ou mais respostas para indicar os tratamentos que conhece. Assinale um círculo “○” à volta do número correspondente.

1	擦傷（如膝蓋擦傷）<br>Escoriações *(como escoriações no joelho)*	5	中暑<br>Insolação
2	肌肉拉傷（如大腿拉傷）<br>Estiramento muscular *(como estiramento das pernas)*	6	運動腹痛（如肚子疼）<br>Dores abdominais *(como dores de barriga)*
3	運動扭傷（如腰扭傷）<br>Entorses *(como entorse lombar)*	7	其他<br>Outros
4	抽筋（如小腿抽筋）<br>Câibras *(como câibras nas pernas)*	8	不知道任何運動損傷的正確處理方法<br>Não tenho conhecimento de nenhum tratamento correcto para lesões desportivas

C5.【單選題】在體育鍛煉中膝蓋擦傷了，最好應該怎麼處理？請在你選中的選項對應的序號上畫“〇”。

【Perguntas de escolha múltipla (com resposta única)】Qual é a melhor forma de tratamento para escoriações sofridas nos joelhos ocorridas durante a prática do exercício físico? Assinale um círculo “〇” à volta do número correspondente.

1	立即用酒精消毒<br>Desinfectar imediatamente com álcool
2	清水沖洗一下即可<br>Lavar com água uma vez
3	清洗後用酒精消毒<br>Desinfectar com álcool, depois de lavar com água
4	其他<br>Outros
5	不知道<br>Não sei

D-健身習慣(由學生獨立回答)
Hábitos de prática de exercício físico *(a preencher pelo aluno)*

D1.【單選題】本學期你平均每週會有多少次上體育課？請在你選中的選項對應的序號上畫“〇”(當兩堂體育課是連著一起上時，也只記為1次；如果不是連著上就記為2次)。

【Perguntas de escolha múltipla (com resposta única)】No presente semestre, quantas vezes por semana frequenta aulas de educação física? Se duas aulas de educação física ocorram consecutivamente, são registadas apenas uma vez; se não ocorram consecutivamente, são registadas como duas aulas distintas. Assinale um círculo "〇" à volta do número correspondente.

1	1次<br>1 vez	4	4次<br>4 vezes
2	2次<br>2 vezes	5	5次或以上<br>5 vezes ou superior
3	3次<br>3 vezes	6	0次(不用回答D2、D3題)<br>Nenhuma vez *(Não necessita responder às perguntas D2 e D3)*

D2.【單選題】你每次上體育課的時間大概是多長？請在你選中的選項對應的序號上畫“〇”。

【Perguntas de escolha múltipla (com resposta única)】Qual é o tempo de duração de cada aula de educação física? Assinale um círculo "〇" à volta do número correspondente.

1	不足30分鐘<br>Inferior a 30 minutos	3	60-89分鐘<br>60 a 89 minutos
2	30-59分鐘<br>30 a 59 minutos	4	90分鐘或以上<br>90 minutos ou superior

D3.【單選題】多數情況下，你上體育課進行體育活動或鍛煉時的身體感受是甚麼樣的？請在你選中的選項對應的序號上畫“〇”。

【Perguntas de escolha múltipla (com resposta única)】Na maioria dos casos, como se sente fisicamente cada vez que pratica exercício físico? Assinale um círculo “〇” à volta do número correspondente

1	呼吸平穩、和平常差不多、沒有特別感覺<br>Respiração lenta, normal, sem qualquer tipo de sensação especial	2	微微出汗、呼吸加快、心跳有點快<br>Transpiração ligeira, respiração acelerada e pulsação cardíaca acelerada um pouco	3	出汗較多、心跳明顯加快、上氣不接下氣<br>Transpiração excessiva, pulsação muito acelerada, ficar sem fôlego.

D4.【單選題】除學校體育課外，最近1個月，你平均每週會有多少次參加體育鍛煉（包括課外體育活動和校外體育鍛煉）？請在你選中的選項對應的序號上畫“〇”。

【Perguntas de escolha múltipla (com resposta única)】Para além das aulas de educação física, quantas vezes em média por semana praticou exercício físico no último mês? (Incluindo actividades desportivas e prática de exercício físico extracurriculares). Assinale um círculo “〇” à volta do número correspondente.

1	平均每週7次或以上<br>Em média 7 vezes ou superior por semana	5	平均每週3次<br>Em média 3 vezes por semana
2	平均每週6次<br>Em média 6 vezes por semana	6	平均每週2次<br>Em média 2 vezes por semana
3	平均每週5次<br>Em média 5 vezes por semana	7	平均每週1次<br>Em média 1 vez por semana
4	平均每週4次<br>Em média 4 vezes por semana	8	0次(不用回答D5、D6題)<br>Nenhuma vez *(Não necessita responder às perguntas D5 e D6)*

D5.【單選題】除學校體育課外，多數情況下，你每次進行體育活動或鍛煉(比如課外體育活動、校外體育鍛煉)的持續時間大概是多長？請在你選中的選項對應的序號上畫“〇”。

【Perguntas de escolha múltipla (com resposta única)】Para além das aulas de educação física, na maioria dos casos, qual foi a duração média das actividades desportivas e do exercício físico praticado? (incluindo actividades desportivas e a prática de exercício físico extracurriculares). Assinale um círculo "〇" à volta do número correspondente.

1	不足30分鐘<br>Inferior a 30 minutos	3	60-89分鐘<br>60 a 89 minutos
2	30-59分鐘<br>30 a 59 minutos	4	90分鐘或以上<br>90 minutos ou superior

D6.【單選題】除學校體育課外，多數情況下，你進行體育活動或鍛煉（比如課外體育活動、校外體育鍛煉）時的身體感受是甚麼樣的？請在你選中的選項對應的序號上畫“○”。

【Perguntas de escolha múltipla (com resposta única)】Para além das aulas de educação física, na maioria dos casos, como se sente fisicamente cada vez que pratica exercício físico? (incluindo actividades desportivas e a prática de exercício físico extracurriculares). Assinale um círculo "〇" à volta do número correspondente.

1	呼吸平穩、和平常差不多、沒有特別感覺<br>Respiração lenta, normal, sem qualquer tipo de sensação especial	2	微微出汗、呼吸加快、心跳有點快<br>Transpiração ligeira, respiração acelerada e pulsação cardíaca acelerada um pouco	3	出汗較多、心跳明顯加快、上氣不接下氣<br>Transpiração excessiva, pulsação muito acelerada, ficar sem fôlego.

D7.【單選題】你通常每週有幾天進行力量練習（包括俯卧撐、仰臥起坐、引體向上、深蹲等徒手練習或使用啞鈴、彈力帶等健身器械的練習）？請在你選中的選項對應的序號上畫“○”。

【Perguntas de escolha múltipla (com resposta única)】Quantos dias por semana pratica exercício físico de força (incluindo exercícios físicos sem equipamentos, nomeadamente flexões de braços, flexões abdominais, apoio frontal sobre as mãos, agachamento profundo e exercícios físicos com equipamentos, nomeadamente halteres e fitas elásticas)? Assinale um círculo “〇” à volta do número correspondente.

1	1天<br>1 dia	3	3天<br>3 dias	5	5天<br>5 dias	7	7天<br>7 dias
2	2天<br>2 dias	4	4天<br>4 dias	6	6天<br>6 dias	8	0天<br>0 dia

D8.【多選題】最近一年，你一直在持續做哪些體育鍛煉（包括體育課、課外體育活動和校外體育鍛煉）？本題爲多選題，可選1個或1個以上的答案，請圈選你一直在持續做的所有體育鍛煉，在選項對應的序號上畫“○”。

【Perguntas de escolha múltipla (com resposta múltipla)】No último ano, quais foram os exercícios físicos que praticou de forma contínua? (incluindo aulas de educação física, actividades desportivas e prática de exercício físico extracurriculares) Esta parte é constituída por perguntas de escolha múltipla, podendo assinalar uma ou mais resposta para indicar os exercícios físicos que praticou de forma continuada. Assinale um círculo "〇" à volta do número correspondente.

1	健步走<br>Caminhada	13	騎自行車<br>Ciclismo	25	水球<br>Pólo aquático
2	中短程跑(1500米內)<br>Corrida de meio-fundo *(1500 metros)*	14	羽毛球<br>Badminton	26	柔道<br>Judo
3	長跑(距離超過1500米)<br>Corrida de fundo *(distância superior a 1500 metros)*	15	網球<br>Ténis	27	滑冰<br>Patinagem no gelo
4	跳高<br>Salto em altura	16	體操<br>Ginástica	28	滑雪<br>Esqui
5	跳遠<br>Salto em comprimento	17	韻律操（形體操、健美操、體育舞蹈、舞蹈等）<br>Ginástica rítmica *(aeróbica, ginástica, dança desportiva, dança etc.)*	29	跳繩<br>Salto à corda
6	籃球<br>Basquetebol	18	游泳<br>Natação	30	踢毽子<br>Shuttlecock *(jogo da peteca)*
7	排球(包括沙灘排球)<br>Voleibol *(Incluindo voleibol de praia)*	19	武術<br>Wushu	31	輪滑<br>Patinagem
8	足球<br>Futebol	20	跆拳道<br>Taekwondo	32	冰球<br>Hóquei no gelo
9	乒乓球<br>Ténis-de-mesa	21	空手道<br>Karate-do	33	舉重<br>Halterofilismo
10	高爾夫球<br>Golfe	22	曲棍球<br>Hóquei	34	標槍<br>Lançamento do dardo
11	棒球<br>Basebol	23	擊劍<br>Esgrima	35	其他<br>Outros
12	鉛球(包括實心球)<br>Lançamento de peso	24	跨欄<br>Corrida com barreiras	36	沒有持續做的體育鍛煉項目(不用回答D9、D10、D11題)<br>Não pratiquei de forma contínua o exercício físico *(Não necessita responder às perguntas D9, D10 e D11)*

D9.【單選題】按上述D8題中選擇的這些體育鍛煉項目，以持續鍛煉最久的項目為主，你大概堅持做了多長時間？請在你選中的選項對應的序號上畫“○”。

【Perguntas de escolha múltipla (com resposta única)】Relativamente à escolha efectuada na pergunta anterior D8, qual foi o tempo mais longo registado na prática dos referidos exercícios físicos? Assinale um círculo "○" à volta do número correspondente.

1	沒有<br>Não consegui manter	3	1個月或以上，但不足3個月<br>Igual ou superior a 1 mês mas, inferior a 3 meses	5	半年或以上，但不足1年<br>Igual ou superior a 6 meses mas, inferior a 1 ano
2	不足1個月<br>Inferior a 1 mês	4	3個月或以上，但不足半年<br>Igual ou superior a 3 meses mas,inferior a 6 meses	6	1年或以上<br>Igual ou superior a 1 ano

D10.【單選題】按上述D8題中選擇的這些體育鍛煉項目，你一直堅持做的主要原因是甚麼？請在你選中的選項對應的序號上畫“○”。

【Perguntas de escolha múltipla (com resposta única)】Relativamente à escolha efectuada na pergunta anterior D8, qual foi o principal motivo que lhe levou praticar de forma contínua esses exercícios físicos? Assinale um círculo "○" à volta do número correspondente.

1	學校或老師要求<br>A pedido da escola ou do professor	3	自己喜歡<br>Pelo interesse próprio	5	其他<br>Outros
2	家長要求<br>A pedido dos pais	4	體育考試或測驗需要<br>Por motivo de realização de exame ou teste físico	6	不知道<br>Não sei

D11.【單選題】你是如何選中D8題中的這些體育鍛煉項目的？請在你選中的選項對應的序號上畫“○”。

【Perguntas de escolha múltipla (com resposta única)】Qual foi o seu critério da escolha dos exercícios físicos referidos na pergunta D8? Assinale com um círculo "○" à volta do número correspondente.

1	自己隨便選的<br>Foi escolhido aleatoriamente	3	跟著學校的要求做的<br>Seguindo os requisitos da escola	5	哪些項目容易就選哪些<br>São actividades desportivas mais fáceis de praticar
2	哪些項目練起來方便就選哪些<br>São actividades desportivas mais convenientes	4	跟著家人、朋友或同學等選的<br>Seguindo a escolha dos familiares, amigos ou colegas de escola	6	諮詢過專業意見<br>De acordo com o conselho profissional

E-其他習慣(可由家長協助回答)
Outros hábitos *(Pode ser preenchido com o auxílio do encarregado de educação)*

E1.【填空題】你通常每天有多少時間坐著或者靠著(包括坐著上文化課、做功課、看電視、使用電腦、吃飯、乘車等等坐著的時間)？

【Preencha os espaços em branco】Quanto tempo por dia, costuma ficar sentado ou encostado? (incluindo estar sentado nas aulas culturais, a fazer trabalhos de casa, a ver televisão, a utilizar o computador, a comer, nos transportes etc.)

請填寫：____ 小時 ____ 分鐘。
Indique: ____ horas ____ minutos.

E2.【單選題】在上學日(週一至週五)，你通常平均每天睡眠時長是多少（含午休）？

【Perguntas de escolha múltipla (com resposta única)】 Durante o período escolar (de segunda a sexta-feira), qual é a duração média de sono, da sua criança (incluindo o intervalo do almoço)?

1	8個小時以下<br>Menos de 8 horas	4	10個小時至不足11個小時<br>10 horas, mas menos do que 11 horas
2	8個小時至不足9個小時<br>8 horas, mas menos do que 9 horas	5	11個小時至不足12個小時<br>11 horas, mas menos do que 12 horas
3	9個小時至不足10個小時<br>9 horas, mas menos do que 10 horas	6	12個小時或以上<br>12 horas ou mais

E3.【單選題】在休息日(週六、週日或節假日)，你通常平均每天睡眠時長是多少（含午休）？

【Perguntas de escolha múltipla (com resposta única)】Durante os dias de descanso (sábado, domingo ou feriados), qual a duração média de sono, da sua criança (incluindo o intervalo do almoço)?

1	8個小時以下<br>Menos de 8 horas	4	10個小時至不足11個小時<br>10 horas, mas menos do que 11 horas
2	8個小時至不足9個小時<br>8 horas, mas menos do que 9 horas	5	11個小時至不足12個小時<br>11 horas, mas menos do que 12 horas
3	9個小時至不足10個小時<br>9 horas, mas menos do que 10 horas	6	12個小時或以上<br>12 horas ou mais

E4.【單選題】在上學日(週一至週五)，你通常平均每天有多長時間花在看電視、使用手機/電腦/平板電腦等電子產品上？請在你選中的選項對應的序號上畫“〇”。

【Perguntas de escolha múltipla (com resposta única)】Durante o período escolar (de segunda a sexta-feira), qual o tempo médio diário utilizado a ver televisão e a utilizar os equipamentos electrónicos, nomeadamente o telemóvel, o computador e o tablete? Assinale um círculo “〇” à volta do número correspondente.

1	1~29分鐘<br>1 a 29 minutos	6	3個小時~不足4個小時<br>3 horas a inferior a 4 horas
2	30~59分鐘<br>30 a 59 minutos	7	4個小時~不足5個小時<br>4 horas a inferior a 5 horas
3	60分鐘-89分鐘<br>60 a 89 minutos	8	5個小時~不足6個小時<br>5 horas a inferior a 6 horas
4	90~119分鐘<br>90 a 119 minutos	9	6個小時或以上<br>6 horais ou superior
5	2個小時~不足3個小時<br>2 horas a inferior a 3 horas	10	一般沒有<br>Nenhum

E5.【單選題】在休息日(週六、週日或節假日)，你通常平均每天有多長時間花在看電視、使用手機/電腦/平板電腦等電子產品上？請在你選中的選項對應的序號上畫“〇”。

【Perguntas de escolha múltipla (com resposta única)】Durante os dias de descanso (sábado, domingo ou feriados), qual o tempo médio diário utilizado a ver televisão e a utilizar os equipamentos electrónicos, nomeadamente o telemóvel, o computador e o tablete? Assinale um círculo “〇” à volta do número correspondente.

1	1~29分鐘<br>1 a 29 minutos	6	3個小時~不足4個小時<br>3 horas a inferior a 4 horas
2	30~59分鐘<br>30 a 59 minutos	7	4個小時~不足5個小時<br>4 horas a inferior a 5 horas
3	60分鐘-89分鐘<br>60 a 89 minutos	8	5個小時~不足6個小時<br>5 horas a inferior a 6 horas
4	90~119分鐘<br>90 a 119 minutos	9	6個小時或以上<br>6 horais ou superior
5	2個小時~不足3個小時<br>2 horas a inferior a 3 horas	10	一般沒有<br>Nenhum

問卷完畢。謝謝。 | Terminou a sua colaboração. Muito obrigado!

三.檢測指標 (由監測人員在監測現場填寫)
Parâmetros de avaliação (a preencher pelo avaliador no local da avaliação)

1.身高（cm）<br>Altura (cm)	□□□.□
2.坐高（cm）<br>Altura medida sentada (cm)	□□.□
3.體重（kg）<br>Peso (kg)	□□□.□
4.胸圍（cm）<br>Circunferência torácica (cm)	□□□.□
5.腰圍（cm）<br>Circunferência abdominal (cm)	□□□.□
6.臀圍（cm）<br>Circunferência do quadril (cm)	□□□.□
7.體脂率（%）<br>Taxa de gordura corporal (%)	□□.□
8.肩寬（cm）<br>Largura dos ombros (cm)	□□.□
9.骨盆寬（cm）<br>Largura da pélvis (cm)	□□.□
10.足長（cm）<br>Comprimento do pé (cm)	□□.□
11.安靜脈搏（次/分）<br>Pulsação em repouso (vezes/minuto)	□□□
12.收縮壓（mmHg）<br>Pressão sistólica (mmHg)	□□□
13.舒張壓（mmHg）<br>Pressão diastólica (mmHg)	□□□

14.肺活量（ml）
Capacidade vital (ml)

15.斜身引體（次）（6~12歲男）
引體向上（次）(13~22歲男)
一分鐘仰臥起坐（次）(6~22歲女)
Elevações em posição inclinado (vezes) (dos 6 aos 12 anos-masculino) /
Elevações (vezes) (dos 13 aos 22 anos-masculino) /
Flexões abdominais durante um minuto (vezes) (dos 6 aos 22 anos-feminino)

16.立定跳遠（cm）
Salto em comprimento sem balanço (cm)

17.50米跑（秒）
Corrida de 50 m (s)

18.50×8往返跑（秒）(6~12歲) /
800米跑（秒） (13~22歲女) /
1000米跑（秒） (13~22歲男)
Corrida de ida e volta de 50 m × 8 (s) (dos 6 aos 12 anos) /
Corrida de 800 m (s) (dos 13 aos 22 anos-feminino)
Corrida de 1000 m (s) (dos 13 aos 22 anos-masculino)

19.握力（kg）
Força de preensão (kg)

20.縱跳（cm）
Salto vertical(cm)

21.背力（kg）
Força dorsal (kg)

22.坐位體前屈（cm）
Flexão ventral sentada (cm)

23.閉眼單腳站立（秒）
Equilíbrio num pé com os olhos fechados (s)

24.選擇反應時（秒）
Velocidade de reacção selectiva (s)

25.齲齒(6～18歲)
Dentes cariados (dos 6 aos 18 anos)

d D m M f F

26.視力
Visão

裸眼視力 Olho nu — 左 esquerdo / 右 direito

串鏡校正 Correcção com lentes — 左 esquerdo: 正片 Positivo; 負片 Negativo

串鏡校正 Correcção com lentes — 右 direito: 正片 Positivo; 負片 Negativo

屈光不正 Ametropia — 左 esquerdo / 右 direito

(0) 正常 Normal (1) 近視 Miopia (2) 遠視 Hipermetropia (3) 其他 Outros

27.色覺檢查
Exame da percepção das cores

(1) 正常 Normal (2) 不正常 Anormal

檢驗員簽署：
Assinatura do avaliador：
